# Associations between pretreatment composite inflammatory indices and pathological response to neoadjuvant therapy in breast cancer: a retrospective cohort study

**DOI:** 10.3389/fonc.2026.1913071

**Published:** 2026-08-17

**Authors:** Yuhong Zhang, Cailing Tong

**Affiliations:** Department of Breast Surgery, The First Affiliated Hospital of Guangzhou University of Chinese Medicine, Guangzhou, China

**Keywords:** breast cancer, Miller–Payne grade, neoadjuvant therapy, pan-immune-inflammation value, pathological complete response, systemic immune-inflammation index

## Abstract

**Background:**

Pretreatment inflammatory indices may reflect the host immune-inflammatory status in breast cancer. Evidence comparing multiple composite indices across pathological response endpoints after neoadjuvant therapy remains limited. This study evaluated the associations between pretreatment composite inflammatory indices and pathological response in breast cancer.

**Methods:**

This retrospective study included 280 patients with breast cancer who underwent neoadjuvant therapy followed by surgery. Baseline neutrophil-to-lymphocyte ratio, platelet-to-lymphocyte ratio, lymphocyte-to-monocyte ratio, systemic immune-inflammation index, systemic inflammation response index, and pan-immune-inflammation value were calculated from pretreatment blood tests. The primary endpoint was pathological complete response, defined as ypT0/is ypN0. Miller-Payne grades 4–5 were defined as favorable pathological response. Logistic regression and receiver operating characteristic analyses were performed.

**Results:**

A total of 115 patients achieved pathological complete response. Patients with pathological complete response had lower neutrophil-to-lymphocyte ratio, systemic immune-inflammation index, and pan-immune-inflammation value than those without pathological complete response. These indices remained inversely associated with pathological complete response after multivariable adjustment. For favorable Miller-Payne response, lower systemic immune-inflammation index and pan-immune-inflammation value and higher lymphocyte-to-monocyte ratio were associated with better response. Systemic immune-inflammation index had the numerically highest area under the curve, with values of 0.673 for pathological complete response and 0.666 for favorable Miller–Payne response, although its discriminatory ability remained modest.

**Conclusion:**

Baseline composite inflammatory indices were associated with pathological response to neoadjuvant therapy in breast cancer, but their discriminatory ability was modest. These indices may be more suitable as supplementary markers than as standalone predictors.

## Introduction

Breast cancer remains one of the most common malignancies worldwide (1). According to the 2022 GLOBOCAN estimates, female breast cancer accounted for approximately 2.3 million new cases and 11.6% of all cancer cases worldwide (2). Neoadjuvant therapy is commonly used for patients with locally advanced breast cancer and for selected patients with high-risk early-stage disease. This treatment strategy can reduce tumor burden before surgery and allows pathological response to be assessed after treatment (3).

Pathological complete response (pCR) is widely used to evaluate the efficacy of neoadjuvant therapy. It is associated with favorable long-term outcomes, especially in triple-negative and HER2-positive breast cancer (4). However, pCR is a binary endpoint and cannot fully describe the degree of tumor regression in patients with residual disease. The Miller–Payne grading system evaluates treatment-induced changes in tumor cellularity by comparing pretreatment core needle biopsy specimens with postoperative surgical specimens (5, 6). Therefore, combined evaluation of pCR and Miller–Payne response may provide a more detailed assessment of pathological response after neoadjuvant therapy.

Treatment response is influenced by both tumor biology and host immune-inflammatory status. In clinical practice, hormone receptor status, HER2 status, Ki-67 index, tumor stage, and treatment regimen are important factors for treatment planning and response assessment (7–9). These factors mainly reflect tumor-related characteristics. Peripheral blood-derived inflammatory indices may provide additional information about systemic inflammation and antitumor immunity.

Previous studies have suggested that inflammatory indices are associated with response to neoadjuvant therapy and prognosis in breast cancer (10–13). However, many studies have focused on one or a limited number of markers. Fewer studies have directly compared multiple composite inflammatory indices in the same cohort using both pCR and Miller–Payne response as pathological endpoints. In addition, whether these associations are consistent across different molecular subtypes remains unclear. This study therefore investigated the associations between pretreatment composite inflammatory indices and pathological response in patients with breast cancer receiving neoadjuvant therapy. We evaluated both pCR and favorable Miller–Payne response, compared the discriminatory ability of several inflammatory indices, and explored whether these associations differed across molecular subtypes.

## Materials and methods

### Study design and patients

**Figure 1:**
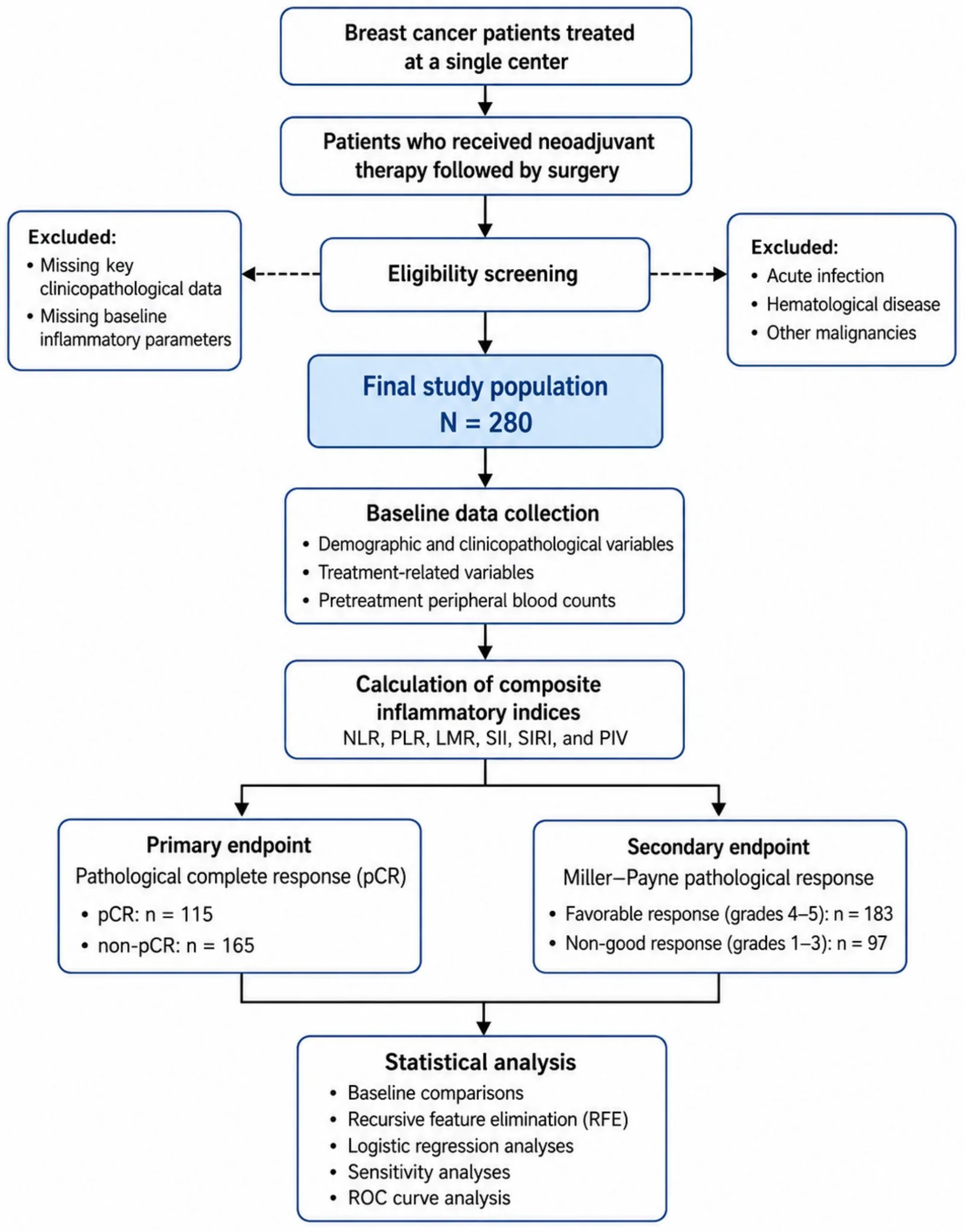
Flowchart of patient selection and study design.

This retrospective observational study included patients with breast cancer who received neoadjuvant therapy followed by surgery at The First Affiliated Hospital of Guangzhou University of Chinese Medicine between January 2021 and January 2026. Patients were screened according to predefined inclusion and exclusion criteria. The study was approved by the institutional ethics committee of The First Affiliated Hospital of Guangzhou University of Chinese Medicine, and the requirement for informed consent was waived because of the retrospective nature of the study.The overall patient selection process and study workflow are shown in **Figure 1**.

### Inclusion and exclusion criteria

#### Inclusion criteria

1. Pathologically confirmed breast cancer.
2. Received neoadjuvant therapy.
3. Completed routine blood tests and biochemical examinations before treatment, allowing calculation of composite inflammatory markers.
4. Underwent surgical resection.
5. Available complete postoperative pathological data for evaluation of pCR and Miller–Payne (MP) grade.

#### Exclusion criteria

1. Presence of other concurrent malignancies.
2. Received other antineoplastic treatments prior to neoadjuvant therapy.
3. Presence of active infection, hematological diseases, or other conditions that may significantly affect inflammatory markers.
4. Missing key clinical, laboratory, or pathological data.

### Definition of molecular subtype

Breast cancer molecular subtypes were classified according to hormone receptor status, HER2 status, and Ki-67 index. Hormone receptor positivity was defined as estrogen receptor or progesterone receptor expression in ≥1% of tumor cells. HER2 positivity was defined as immunohistochemistry 3+ or fluorescence *in situ* hybridization-confirmed HER2 amplification in cases with immunohistochemistry 2 +. Patients were categorized into luminal, HER2-positive, and triple-negative breast cancer subgroups according to clinicopathological criteria.

### Blood sampling and inflammatory markers

Peripheral blood samples were obtained after diagnostic core needle biopsy and within 7 days before initiation of neoadjuvant therapy. All blood samples were collected before any systemic antitumor treatment.

Complete blood count parameters, including neutrophil count, lymphocyte count, monocyte count, and platelet count, were collected. All blood samples were acquired prior to any systemic antitumor treatment. Patients with clinical evidence of acute infection or active inflammatory disease at the time of blood sampling were excluded.

The following composite inflammatory markers were calculated:

NLR = neutrophil count/lymphocyte count
PLR = platelet count/lymphocyte count
LMR = lymphocyte count/monocyte count
SII = (platelet count × neutrophil count)/lymphocyte count
SIRI = (neutrophil count × monocyte count)/lymphocyte count
PIV = (neutrophil count × platelet count × monocyte count)/lymphocyte count

Patients with clinical evidence of acute infection, active inflammatory disease, hematological disease, or other conditions that could substantially affect peripheral blood cell counts were excluded. These conditions were identified through medical records, clinical symptoms, body temperature, laboratory findings, medication history, and physician documentation before treatment.

### Definition of pathological response

Pathological complete response was defined as the absence of residual invasive carcinoma in the breast and axillary lymph nodes after neoadjuvant therapy, regardless of the presence of residual ductal carcinoma *in situ*, corresponding to ypT0/is ypN0.

For subsequent statistical analyses, patients with Miller–Payne grades 4–5 were classified as having a favorable pathological response, because these grades indicate marked histological response, defined as more than 90% reduction in invasive tumor cellularity or no identifiable invasive malignant cells in the primary tumor bed. In contrast, grades 1–3 indicate absent, mild, or moderate reduction in tumor cellularity and were therefore classified as non-favorable pathological response (6).

### Definition of HER2 status

HER2 status was determined according to immunohistochemistry and fluorescence *in situ* hybridization results. HER2 positivity was defined as immunohistochemistry 3+ or immunohistochemistry 2+ with fluorescence *in situ* hybridization-confirmed HER2 amplification. Tumors with immunohistochemistry 2+ but without HER2 amplification were classified as HER2-negative.

### Statistical analysis

Continuous variables were expressed as mean ± standard deviation (SD) for normally distributed data or as median (interquartile range, IQR) for non-normally distributed data. Categorical variables were summarized as counts (%) accordingly. Data normality was evaluated using the Kolmogorov–Smirnov test. Between-group differences were examined using Student’s t test for normally distributed variables and the Mann–Whitney U test for skewed data. For categorical comparisons, the Chi-squared test was applied unless the expected frequency in any cell was < 5, in which case Fisher’s exact test was used.

To identify candidate variables associated with pathological response to neoadjuvant therapy in breast cancer, recursive feature elimination (RFE) was performed before multivariable logistic regression analysis. RFE was conducted using a random forest algorithm as the base estimator. A 5-fold cross-validation procedure was applied to evaluate model performance across different variable subsets. The optimal number of variables was determined according to the variable subset with the highest cross-validated accuracy. Separate RFE procedures were performed for pathological complete response (pCR) and favorable Miller–Payne pathological response. Variables retained by RFE were then used to guide the selection of covariates for the subsequent multivariable logistic regression models. Variable names were standardized across the baseline characteristics tables, RFE output, and regression analyses to ensure consistency. Subsequently, multivariable logistic regression analysis was performed to evaluate the associations of PLR, NLR, SII, SIRI, PIV, and LMR with pathologic response to neoadjuvant therapy in breast cancer. For logistic regression analyses, all inflammatory indices were analyzed as continuous variables after Z-score standardization. Therefore, the odds ratios represent the change in the odds of pathological response per one-standard-deviation increase in each inflammatory index.

For each pathological endpoint, two logistic regression models were fitted separately. Model 1 was an unadjusted model. For pathological complete response, Model 2 was adjusted for menopausal status, ER status, HER2 status, clinical T stage, clinical N stage, platinum-containing therapy, anti-HER2 therapy, Ki-67 index, number of metastatic lymph nodes, and age. For favorable Miller–Payne pathological response, Model 2 was adjusted for HER2 status, anti-HER2 therapy, Ki-67 index, number of metastatic lymph nodes, BMI, menopausal status, clinical N stage, clinical T stage, age, and ER status. Multicollinearity among the variables included in the adjusted models was assessed using variance inflation factors. A VIF <5 was considered to indicate the absence of substantial multicollinearity.

To test the robustness of our findings, several sensitivity analyses were performed: (1) for pCR, BMI and PR status were added to the covariates included in Model 2, whereas for favorable Miller–Payne response, platinum-containing therapy was added to the covariates included in Model 2; (2) age-stratified analyses were conducted for patients aged <60 years and ≥60 years, together with formal interaction analyses to evaluate whether age modified the associations between inflammatory indices and pathological response; and (3) the composite inflammatory indices were converted from continuous variables into categorical variables. In the categorical sensitivity analyses, inflammatory indices were dichotomized according to their median values and reanalyzed as categorical variables.

The discriminatory ability of all composite inflammatory indices was evaluated and compared using receiver operating characteristic (ROC) curve analysis, with the area under the curve (AUC) and its 95% confidence interval (CI) reported. All statistical analyses were performed using R software, version [4.4.2]. RFE was implemented using the caret package, with random forest used as the base estimator. Logistic regression analyses were performed using the glm function in the stats package, and ROC analyses were conducted using the pROC package. Bonferroni correction was applied separately to the six primary inflammatory-index comparisons within each pathological endpoint, with an adjusted P value <0.05 considered statistically significant. A two-sided P value <0.05 was considered statistically significant.

## Results

### Baseline characteristics

A total of 280 patients were included in this retrospective analysis. The demographic, clinical, pathological, inflammatory, and treatment-related characteristics of the cohort, stratified by pathological complete response (pCR) status and Miller–Payne (MP) pathological response, are presented in **Tables 1**, **2**, respectively.

**Table 1:** Patient characteristics stratified by pathological complete response status.

Characteristic	pCR			P-value
	Overall N = 280	No N = 165	Yes N = 115	
**PLR, Median (Q1, Q3)**	156 (123, 192)	155 (124, 191)	158 (121, 193)	0.937^1^
**NLR, Median (Q1, Q3)**	2.20 (1.71, 2.65)	2.37 (1.83, 2.78)	2.02 (1.58, 2.41)	<0.001^1^
**LMR, Median (Q1, Q3)**	4.43 (3.35, 5.61)	4.32 (3.21, 5.52)	4.55 (3.54, 5.72)	0.124^1^
**SII, Median (Q1, Q3)**	608 (432, 829)	667 (484, 952)	527 (381, 705)	<0.001^1^
**SIRI, Median (Q1, Q3)**	0.89 (0.58, 1.26)	0.85 (0.57, 1.33)	0.91 (0.58, 1.18)	0.405^1^
**PIV, Median (Q1, Q3)**	288 (196, 408)	311 (223, 448)	248 (164, 356)	<0.001^1^
**Ki-67, Median (Q1, Q3)**	49 (31, 65)	45 (30, 60)	55 (35, 70)	0.003^1^
**Number of metastatic lymph nodes, Median (Q1, Q3)**	0.00 (0.00, 1.00)	0.00 (0.00, 1.00)	0.00 (0.00, 1.00)	0.801^1^
**Age, Median (Q1, Q3)**	50 (42, 57)	51 (43, 58)	48 (40, 55)	0.017^1^
**BMI, Median (Q1, Q3)**	23.5 (21.4, 26.1)	23.5 (21.5, 26.1)	23.4 (21.3, 25.9)	0.715^1^
**menopause, n (%)**	119 (42.5%)	62 (37.6%)	57 (49.6%)	0.046^2^
**Molecular subtype, n (%)**				0.001^2^
Luminal	112 (40.0%)	80 (48.5%)	32 (27.8%)	
HER2-positive	124 (44.3%)	66 (40.0%)	58 (50.4%)	
Triple-negative breast cancer	44 (15.7%)	19 (11.5%)	25 (21.7%)	
**ER, n (%)**	162 (57.9%)	103 (62.4%)	59 (51.3%)	0.064^2^
**PR, n (%)**	109 (38.9%)	70 (42.4%)	39 (33.9%)	0.151^2^
**HER2, n (%)**	126 (45.0%)	66 (40.0%)	60 (52.2%)	0.044^2^
**Neoadjuvant chemotherapy regimen, n (%)**				
Anthracycline-taxane (EC-T/AC-T)	123 (43.9%)	90 (54.5%)	33 (28.7%)	
Taxane-based (TC/THP/TCbHP)	125 (44.6%)	61 (37.0%)	64 (55.7%)	
Other regimens	32 (11.4%)	14 (8.5%)	18 (15.7%)	
**cT, n (%)**				0.479^2^
cT1	15 (5.4%)	8 (4.8%)	7 (6.1%)	
cT2	209 (74.6%)	119 (72.1%)	90 (78.3%)	
cT3	39 (13.9%)	27 (16.4%)	12 (10.4%)	
cT4	17 (6.1%)	11 (6.7%)	6 (5.2%)	
**cN, n (%)**				0.317^2^
cN0	53 (18.9%)	26 (15.8%)	27 (23.5%)	
cN1	164 (58.6%)	98 (59.4%)	66 (57.4%)	
cN3	32 (11.4%)	22 (13.3%)	10 (8.7%)	
cN2	31 (11.1%)	19 (11.5%)	12 (10.4%)	
**Platinum-containing treatment, n (%)**	119 (42.5%)	65 (39.4%)	54 (47.0%)	0.208^2^
**Anti-HER2 therapy, n (%)**	123 (43.9%)	62 (37.6%)	61 (53.0%)	0.010^2^
**Surgical-method, n (%)**				0.991^2^
Modified radical mastectomy	172 (61.4%)	101 (61.2%)	71 (61.7%)	
Breast-conserving surgery	62 (22.1%)	37 (22.4%)	25 (21.7%)	
Simple mastectomy	46 (16.4%)	27 (16.4%)	19 (16.5%)	
**Miller-Payne favorable response, n (%)**	183 (65.4%)	68 (41.2%)	115 (100.0%)	

^1^ Wilcoxon rank sum test.

^2^ Pearson’s Chi-squared test.

^3^ Fisher’s exact test.

Bold values indicate statistical significance (P < 0.05).

**Table 2:** Patient characteristics stratified by Miller–Payne pathological response.

Characteristic	Miller_Payne			P-value
	Overall N = 280	Favorable response N = 183	Non-favorable response N = 97	
**PLR, Median (Q1, Q3)**	156 (123, 192)	157 (121, 195)	156 (128, 188)	0.885^1^
**NLR, Median (Q1, Q3)**	2.20 (1.71, 2.65)	2.15 (1.70, 2.58)	2.32 (1.74, 2.74)	0.137^1^
**LMR, Median (Q1, Q3)**	4.43 (3.35, 5.61)	4.67 (3.61, 5.77)	3.97 (2.96, 5.28)	0.003^1^
**SII, Median (Q1, Q3)**	608 (432, 829)	555 (407, 747)	743 (520, 984)	<0.001^1^
**SIRI, Median (Q1, Q3)**	0.89 (0.58, 1.26)	0.88 (0.60, 1.22)	0.89 (0.55, 1.32)	0.580^1^
**PIV, Median (Q1, Q3)**	288 (196, 408)	270 (179, 390)	305 (213, 435)	0.019^1^
**Ki-67, Median (Q1, Q3)**	49 (31, 65)	51 (35, 67)	45 (27, 62)	0.031^1^
**Number of metastatic lymph nodes, Median (Q1, Q3)**	0.00 (0.00, 1.00)	0.00 (0.00, 1.00)	0.00 (0.00, 2.00)	0.321^1^
**Age, Median (Q1, Q3)**	50 (42, 57)	49 (41, 57)	51 (43, 57)	0.237^1^
**BMI, Median (Q1, Q3)**	23.5 (21.4, 26.1)	23.6 (21.5, 25.9)	23.3 (20.4, 26.2)	0.781^1^
**menopause, n (%)**	119 (42.5%)	76 (41.5%)	43 (44.3%)	0.652^2^
**Molecular subtype, n (%)**				0.066^2^
Luminal	112 (40.0%)	67 (36.6%)	45 (46.4%)	
HER2-positive	124 (44.3%)	81 (44.3%)	43 (44.3%)	
Triple-negative breast cancer	44 (15.7%)	35 (19.1%)	9 (9.3%)	
Neoadjuvant chemotherapy regimen				
Anthracycline-taxane EC-T/AC-T	123 (43.9%)	67 (36.6%)	56 (57.7%)	
Taxane-based TC/THP/TCbHP	125 (44.6%)	90 (49.2%)	35 (36.1%)	
Other regimens	32 (11.4%)	26 (14.2%)	6 (6.2%)	
**ER, n (%)**	162 (57.9%)	104 (56.8%)	58 (59.8%)	0.633^2^
**PR, n (%)**	109 (38.9%)	71 (38.8%)	38 (39.2%)	0.951^2^
**HER2, n (%)**	126 (45.0%)	92 (50.3%)	34 (35.1%)	0.015^2^
**cT, n (%)**				0.878^2^
cT1	15 (5.4%)	11 (6.0%)	4 (4.1%)	
cT2	209 (74.6%)	137 (74.9%)	72 (74.2%)	
cT3	39 (13.9%)	24 (13.1%)	15 (15.5%)	
cT4	17 (6.1%)	11 (6.0%)	6 (6.2%)	
**cN, n (%)**				0.561^2^
cN0	53 (18.9%)	39 (21.3%)	14 (14.4%)	
cN1	164 (58.6%)	105 (57.4%)	59 (60.8%)	
cN2	31 (11.1%)	19 (10.4%)	12 (12.4%)	
cN3	32 (11.4%)	20 (10.9%)	12 (12.4%)	
**Platinum-containing treatment, n (%)**	119 (42.5%)	80 (43.7%)	39 (40.2%)	0.572^2^
**Anti-HER2 therapy, n (%)**	123 (43.9%)	93 (50.8%)	30 (30.9%)	0.001^2^
**Surgical method, n (%)**				0.565^2^
Modified radical mastectomy	172 (61.4%)	109 (59.6%)	63 (64.9%)	
Breast-conserving surgery	62 (22.1%)	41 (22.4%)	21 (21.6%)	
Simple mastectomy	46 (16.4%)	33 (18.0%)	13 (13.4%)	
**pCR, n (%)**	115 (41.1%)	115 (62.8%)	0 (0%)	<0.001^2^

^1^ Wilcoxon rank sum test.

^2^ Pearson’s Chi-squared test.

^3^ Fisher’s exact test.

Bold values indicate statistical significance (P < 0.05).

### Patient characteristics stratified by pathological complete response status

Among the 280 patients included in the analysis, 115 (41.1%) achieved pCR. Compared with patients who did not achieve pCR, those who achieved pCR had lower NLR, SII, and PIV levels. Several clinicopathological and treatment-related characteristics, including age, menopausal status, molecular subtype, Ki-67 index, HER2 status, and receipt of anti-HER2 therapy, also differed between the two groups. Detailed patient characteristics are presented in **Table 1**.

### Patient characteristics stratified by Miller-Payne pathological response grade

Among the 280 patients, 183 (65.4%) achieved a favorable Miller–Payne response, whereas 97 (34.6%) had a non-favorable response. Compared with patients with a non-favorable response, those with a favorable response had higher LMR and lower SII and PIV levels. Ki-67 index, HER2 status, and receipt of anti-HER2 therapy also differed between the two groups. Detailed patient characteristics are presented in **Table 2**.

### Exploratory recursive feature elimination

Recursive feature elimination (RFE) was applied as an exploratory feature selection method to identify variables potentially associated with pathological complete response (pCR) and favorable pathological response in patients with breast cancer undergoing neoadjuvant therapy. In the RFE output, variables highlighted in red were selected by the algorithm, whereas those highlighted in green were not selected.

**Figure 2:**
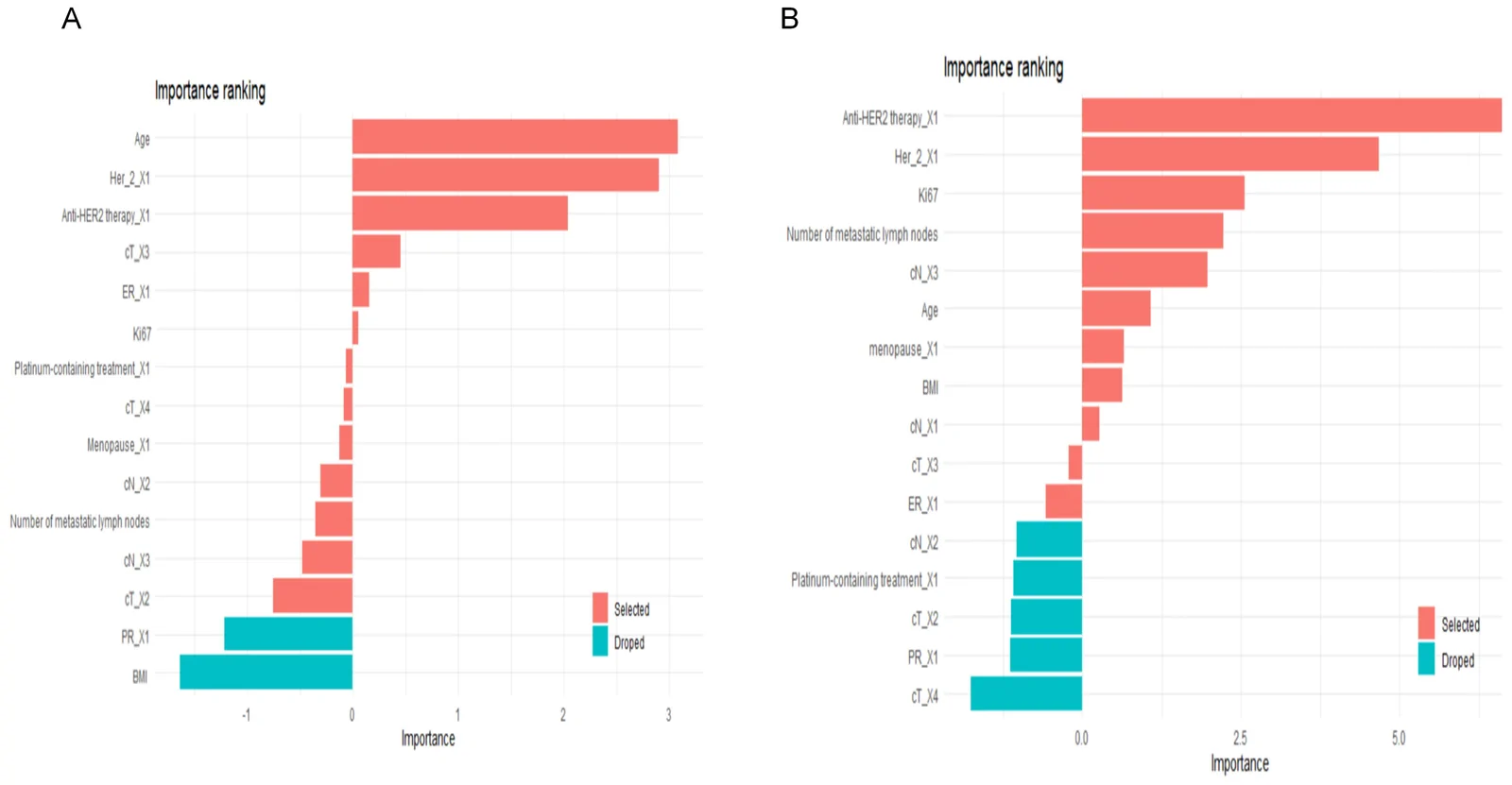
Recursive feature elimination analysis for pathological response endpoints. **(A)** Feature importance ranking for pathological complete response. **(B)** Feature importance ranking for favorable Miller–Payne pathological response. Red bars indicate variables selected by the RFE algorithm, whereas green bars indicate variables not selected. pCR, pathological complete response; RFE, recursive feature elimination.

For pCR, RFE selected 10 potentially relevant features from the candidate variables: menopausal status, ER status, HER2 status, clinical T stage, clinical N stage, platinum-containing therapy, anti-HER2 therapy, Ki-67 index, number of metastatic lymph nodes, and age. For favorable pathological response, RFE selected HER2 status, anti-HER2 therapy, Ki-67 index, Number of metastatic lymph nodes, BMI, menopause, clinical N stage, clinical T stage, age, and ER status. The RFE feature importance rankings for pCR and favorable Miller–Payne response are shown in **Figures 2A, B**, respectively. The variables retained by RFE were used to guide covariate selection in the subsequent multivariable logistic regression models.

### The association between pretreatment inflammatory indices and pathological complete response

The results of logistic regression analyses examining the association between pretreatment inflammatory indices and pathological complete response (pCR) are presented in **Table 3**. In the unadjusted model, NLR, SII, and PIV were significantly and inversely associated with the likelihood of achieving pCR. Higher NLR was associated with lower odds of pCR (OR = 0.57; 95% CI: 0.44–0.73; P<0.001). Similar inverse associations were observed for SII (OR = 0.47; 95% CI: 0.35–0.62; P<0.001) and PIV (OR = 0.54; 95% CI: 0.41–0.71; P<0.001). In contrast, PLR, SIRI, and LMR were not significantly associated with pCR in the unadjusted model (PLR: OR = 0.99, P = 0.909; SIRI: OR = 0.85, P = 0.177; LMR: OR = 1.22, P = 0.111).

**Table 3:** Logistic regression analysis of composite inflammatory indices for pathological complete response.

Variable	Model 1		Model 2		Bonferroni-adjusted P-value
	OR (95% CI)	P-value	OR (95% CI)	P-value	
PLR	0.99 (0.78, 1.25)	0.909	0.94 (0.72, 1.22)	0.628	0.999
NLR	0.57 (0.44, 0.73)	<0.001	0.59 (0.45, 0.79)	<0.001	0.002
LMR	1.22 (0.96, 1.55)	0.111	1.25 (0.95, 1.63)	0.109	0.653
SII	0.47 (0.35, 0.62)	<0.001	0.45 (0.33, 0.62)	<0.001	<0.001
SIRI	0.85 (0.67, 1.08)	0.177	0.87 (0.67, 1.14)	0.322	0.999
PIV	0.54 (0.41, 0.71)	<0.001	0.46 (0.34, 0.63)	<0.001	<0.001

OR, odds ratio; CI, confidence interval.

ORs for continuous inflammatory indices are reported per one-standard-deviation increase after Z-score standardization.

Model 1: unadjusted model.

Model 2: adjusted for menopausal status, ER status, HER2 status, clinical T stage, clinical N stage, platinum-containing therapy, anti-HER2 therapy, Ki-67 index, number of metastatic lymph nodes, and age.

Bonferroni correction was applied to the six Model 2 comparisons within this endpoint.

After adjustment for menopausal status, ER status, HER2 status, clinical T stage, clinical N stage, platinum-containing therapy, anti-HER2 therapy, Ki-67 index, number of metastatic lymph nodes, and age, NLR, SII, and PIV remained significantly and inversely associated with pCR. The adjusted ORs were 0.59 for NLR (95% CI: 0.45–0.79; P<0.001), 0.45 for SII (95% CI: 0.33–0.62; P<0.001), and 0.46 for PIV (95% CI: 0.34–0.63; P<0.001). No significant associations were observed for PLR (OR = 0.94; 95% CI: 0.72–1.22; P = 0.628), SIRI (OR = 0.87; 95% CI: 0.67–1.14; P = 0.322), or LMR (OR = 1.25; 95% CI: 0.95–1.63; P = 0.109). After Bonferroni correction, NLR, SII, and PIV remained statistically significant, with adjusted P values of 0.002, <0.001, and <0.001, respectively.

### The association between pretreatment inflammatory indices and a favorable pathological response

The results of the logistic regression analyses are presented in **Table 4**. The association between pretreatment inflammatory indices and favorable pathological response was evaluated using univariable and multivariable logistic regression analyses. In the unadjusted model, PLR (OR = 0.98; 95% CI: 0.77–1.26; P = 0.886), NLR (OR = 0.82; 95% CI: 0.64–1.05; P = 0.122), and SIRI (OR = 0.88; 95% CI: 0.69–1.13; P = 0.326) were not significantly associated with favorable pathological response. In contrast, SII (OR = 0.52; 95% CI: 0.40–0.68; P<0.001), PIV (OR = 0.74; 95% CI: 0.58–0.95; P = 0.016), and LMR (OR = 1.47; 95% CI: 1.14–1.90; P = 0.003) showed significant associations.

**Table 4:** Logistic regression analysis of composite inflammatory indices for favorable Miller–Payne pathological response.

Variable	Model 1		Model 2		Bonferroni-adjusted P-value
	OR (95% CI)	P-value	OR (95% CI)	P-value	
PLR	0.98 (0.77, 1.26)	0.886	0.92 (0.71, 1.21)	0.558	0.999
NLR	0.82 (0.64, 1.05)	0.122	0.89 (0.68, 1.17)	0.416	0.999
LMR	1.47 (1.14, 1.90)	0.003	1.54 (1.17, 2.04)	0.002	0.014
SII	0.52 (0.40, 0.68)	<0.001	0.54 (0.41, 0.71)	<0.001	<0.001
SIRI	0.88 (0.69, 1.13)	0.326	0.92 (0.71, 1.20)	0.525	0.999
PIV	0.74 (0.58, 0.95)	0.016	0.69 (0.53, 0.90)	0.007	0.041

OR, odds ratio; CI, confidence interval.

ORs for continuous inflammatory indices are reported per one-standard-deviation increase after Z-score standardization.

Model 1: unadjusted model.

Model 2: adjusted for HER2 status, anti-HER2 therapy, Ki-67 index, number of metastatic lymph nodes, BMI, menopausal status, clinical N stage, clinical T stage, age, and ER status.

Bonferroni correction was applied to the six Model 2 comparisons within this endpoint.

In the multivariable models adjusted for HER2 status, anti-HER2 therapy, Ki-67 index, number of metastatic lymph nodes, BMI, menopausal status, clinical N stage, clinical T stage, age, and ER status, SII, PIV, and LMR remained significantly associated with favorable pathological response. The adjusted ORs were 0.54 for SII (95% CI: 0.41–0.71; P<0.001), 0.69 for PIV (95% CI: 0.53–0.90; P = 0.007), and 1.54 for LMR (95% CI: 1.17–2.04; P = 0.002). PLR (OR = 0.92; 95% CI: 0.71–1.21; P = 0.558), NLR (OR = 0.89; 95% CI: 0.68–1.17; P = 0.416), and SIRI (OR = 0.92; 95% CI: 0.71–1.20; P = 0.525) remained non-significant. After Bonferroni correction, LMR, SII, and PIV remained statistically significant, with adjusted P values of 0.014, <0.001, and 0.041, respectively. Multicollinearity diagnostics identified no substantial collinearity, with maximum VIF values of 1.177 in the pCR models and 1.202 in the favorable Miller–Payne response models.

### Sensitivity analysis

#### Pretreatment inflammatory indices and pathological complete response

With additional adjustment for BMI and PR, NLR (OR = 0.58; 95% CI: 0.44–0.78; P<0.001), SII (OR = 0.44; 95% CI: 0.32–0.61; P<0.001), and PIV (OR = 0.46; 95% CI: 0.34–0.62; P<0.001) remained inversely associated with pCR. PLR (OR = 0.90; 95% CI: 0.68–1.18; P = 0.432), SIRI (OR = 0.85; 95% CI: 0.65–1.12; P = 0.250), and LMR (OR = 1.31; 95% CI: 1.00–1.73; P = 0.053) were not statistically significant.

In the age-stratified analyses, NLR (OR = 0.62; 95% CI: 0.45–0.84; P = 0.003), SII (OR = 0.48; 95% CI: 0.34–0.69; P<0.001), and PIV (OR = 0.48; 95% CI: 0.34–0.67; P<0.001) were significantly associated with pCR among patients aged <60 years. Among patients aged ≥60 years, significant inverse associations were observed for PLR (OR = 0.29; 95% CI: 0.10–0.80; P = 0.018), NLR (OR = 0.28; 95% CI: 0.09–0.92; P = 0.035), and SII (OR = 0.16; 95% CI: 0.04–0.65; P = 0.010). Formal interaction analysis identified a significant interaction only for PLR (P for interaction=0.005); the interaction terms for the other indices were not statistically significant (**Supplementary Table 1**).

When the inflammatory indices were dichotomized at their overall medians, high NLR (OR = 0.46; 95% CI: 0.27–0.78; P = 0.004), high SII (OR = 0.40; 95% CI: 0.23–0.68; P<0.001), and high PIV (OR = 0.45; 95% CI: 0.26–0.77; P = 0.004) were associated with lower odds of pCR. Dichotomized PLR, LMR, and SIRI were not significantly associated with pCR.

#### Pretreatment inflammatory indices and favorable pathological response

After additional adjustment for platinum-containing therapy, LMR remained positively associated with favorable pathological response (OR = 1.54; 95% CI: 1.16–2.03; P = 0.002), whereas SII (OR = 0.53; 95% CI: 0.40–0.71; P<0.001) and PIV (OR = 0.69; 95% CI: 0.52–0.90; P = 0.006) remained inversely associated with the outcome. No significant associations were observed for PLR, NLR, or SIRI.

In patients aged <60 years, LMR was positively associated with favorable pathological response (OR = 1.57; 95% CI: 1.13–2.17; P = 0.007), whereas SII (OR = 0.54; 95% CI: 0.40–0.74; P<0.001) and PIV (OR = 0.62; 95% CI: 0.46–0.84; P = 0.002) were inversely associated with the outcome. Among patients aged ≥60 years, significant inverse associations were observed for PLR (OR = 0.36; 95% CI: 0.15–0.92; P = 0.032) and NLR (OR = 0.21; 95% CI: 0.07–0.67; P = 0.008). Formal interaction analysis identified a significant interaction only for PLR (P for interaction=0.006); the remaining interaction terms were not statistically significant (**Supplementary Table 1**).

When the inflammatory indices were dichotomized at their overall medians, high LMR was associated with higher odds of favorable pathological response (OR = 2.16; 95% CI: 1.24–3.76; P = 0.006), whereas high SII was associated with lower odds of favorable response (OR = 0.41; 95% CI: 0.24–0.71; P = 0.001). Dichotomized PLR, NLR, SIRI, and PIV were not significantly associated with this outcome. Detailed sensitivity-analysis results are presented in **Table 5**.

**Table 5:** Sensitivity analyses of composite inflammatory indices for pathological response endpoints.

Characteristics	OR (95% CI)	P-value	
Pathological complete response			
Additional adjustment for BMI and PR			
PLR	0.90 (0.68, 1.18)		0.432
NLR	0.58 (0.44, 0.78)		<0.001
LMR	1.31 (1.00, 1.73)		0.053
SII	0.44 (0.32, 0.61)		<0.001
SIRI	0.85 (0.65, 1.12)		0.250
PIV	0.46 (0.34, 0.62)		<0.001
Subgroup analyses by age (<60 years vs. ≥60 years)			
PLR			
<60 years	1.14 (0.84, 1.54)		0.393
≥60 years	0.29 (0.10, 0.80)		0.018
NLR			
<60 years	0.62 (0.45, 0.84)		0.003
≥60 years	0.28 (0.09, 0.92)		0.035
LMR			
<60 years	1.18 (0.87, 1.60)		0.287
≥60 years	1.64 (0.74, 3.63)		0.225
SII			
<60 years	0.48 (0.34, 0.69)		<0.001
≥60 years	0.16 (0.04, 0.65)		0.010
SIRI			
<60 years	0.93 (0.68, 1.27)		0.654
≥60 years	0.68 (0.30, 1.53)		0.352
PIV			
<60 years	0.48 (0.34, 0.67)		<0.001
≥60 years	0.53 (0.20, 1.39)		0.196
Inflammatory indices dichotomized at their overall medians			
PLR			
Low (<156.31)	Reference		
High (≥156.31)	1.03 (0.61, 1.74)		0.918
NLR			
Low (<2.195)	Reference		
High (≥2.195)	0.46 (0.27, 0.78)		0.004
LMR			
Low (<4.425)	Reference		
High (≥4.425)	1.38 (0.82, 2.34)		0.226
SII			
Low (<607.67)	Reference		
High (≥607.67)	0.40 (0.23, 0.68)		<0.001
SIRI			
Low (<0.885)	Reference		
High (≥0.885)	1.32 (0.78, 2.24)		0.307
PIV			
Low (<288.15)	Reference		
High (≥288.15)	0.45 (0.26, 0.77)		0.004
Favorable Miller–Payne response			
Additional adjustment for platinum-containing therapy			
PLR	0.92 (0.70, 1.20)		0.532
NLR	0.90 (0.69, 1.17)		0.421
LMR	1.54 (1.16, 2.03)		0.002
SII	0.53 (0.40, 0.71)		<0.001
SIRI	0.92 (0.71, 1.20)		0.530
PIV	0.69 (0.52, 0.90)		0.006
Subgroup analyses by age (<60 years vs. ≥60 years)			
PLR			
<60 years	1.09 (0.80, 1.49)		0.565
≥60 years	0.36 (0.15, 0.92)		0.032
NLR			
<60 years	1.01 (0.75, 1.37)		0.931
≥60 years	0.21 (0.07, 0.67)		0.008
LMR			
<60 years	1.57 (1.13, 2.17)		0.007
≥60 years	2.24 (0.97, 5.16)		0.058
SII			
<60 years	0.54 (0.40, 0.74)		<0.001
≥60 years	0.40 (0.14, 1.11)		0.078
SIRI			
<60 years	0.90 (0.66, 1.23)		0.507
≥60 years	0.81 (0.39, 1.68)		0.573
PIV			
<60 years	0.62 (0.46, 0.84)		0.002
≥60 years	0.89 (0.35, 2.23)		0.796
Inflammatory indices dichotomized at their overall medians			
PLR			
Low (<156.31)	Reference		
High (≥156.31)	0.95 (0.55, 1.63)		0.841
NLR			
Low (<2.195)	Reference		
High (≥2.195)	0.82 (0.48, 1.39)		0.456
LMR			
Low (<4.425)	Reference		
High (≥4.425)	2.16 (1.24, 3.76)		0.006
SII			
Low (<607.67)	Reference		
High (≥607.67)	0.41 (0.24, 0.71)		0.001
SIRI			
Low (<0.885)	Reference		
High (≥0.885)	1.05 (0.62, 1.79)		0.853
PIV			
Low (<288.15)	Reference		
High (≥288.15)	0.67 (0.39, 1.13)		0.136

For the pCR sensitivity analyses, the additional-adjustment model included BMI and PR in addition to the revised pCR covariates. For favorable Miller–Payne response, platinum-containing therapy was added to the revised model. Age-stratified models included the corresponding revised covariates except continuous age. Median-dichotomized models used the low group as the reference. Sensitivity and subgroup P values were not adjusted for multiple comparisons.

ORs for continuous inflammatory indices are reported per one-standard-deviation increase after Z-score standardization. For dichotomized analyses, the low group was used as the reference category.

### Subgroup analyses of pretreatment inflammatory indices for favorable pathological response and pathological complete response

For pCR, NLR was inversely associated with the outcome in the luminal (OR = 0.53; 95% CI: 0.35–0.80; P = 0.003) and HER2-positive subgroups (OR = 0.57; 95% CI: 0.38–0.86; P = 0.006). SII was significantly associated with pCR in the luminal (OR = 0.58; 95% CI: 0.37–0.91; P = 0.018), HER2-positive (OR = 0.43; 95% CI: 0.27–0.66; P<0.001), and TNBC subgroups (OR = 0.36; 95% CI: 0.17–0.78; P = 0.009), while PIV was significant in the HER2-positive (OR = 0.38; 95% CI: 0.25–0.58; P<0.001) and TNBC subgroups (OR = 0.30; 95% CI: 0.13–0.70; P = 0.005). For favorable pathological response, LMR was significant in the luminal (OR = 1.75; 95% CI: 1.14–2.69; P = 0.011) and HER2-positive subgroups (OR = 1.79; 95% CI: 1.20–2.67; P = 0.004). Significant associations were also observed for SII in the HER2-positive (OR = 0.36; 95% CI: 0.23–0.57; P<0.001) and TNBC subgroups (OR = 0.41; 95% CI: 0.17–0.97; P = 0.043), SIRI in the TNBC subgroup (OR = 3.77; 95% CI: 1.21–11.76; P = 0.022), and PIV in the HER2-positive (OR = 0.66; 95% CI: 0.46–0.95; P = 0.023) and TNBC subgroups (OR = 0.32; 95% CI: 0.11–0.90; P = 0.032).

Detailed molecular subtype results are presented in **Supplementary Table 2**. Because the fully adjusted models were unstable in the TNBC subgroup owing to its limited sample size, the subtype-stratified estimates were obtained from marker-only logistic regression models and should be regarded as exploratory.

### Discriminatory ability of pretreatment inflammatory indices

**Figure 3:**
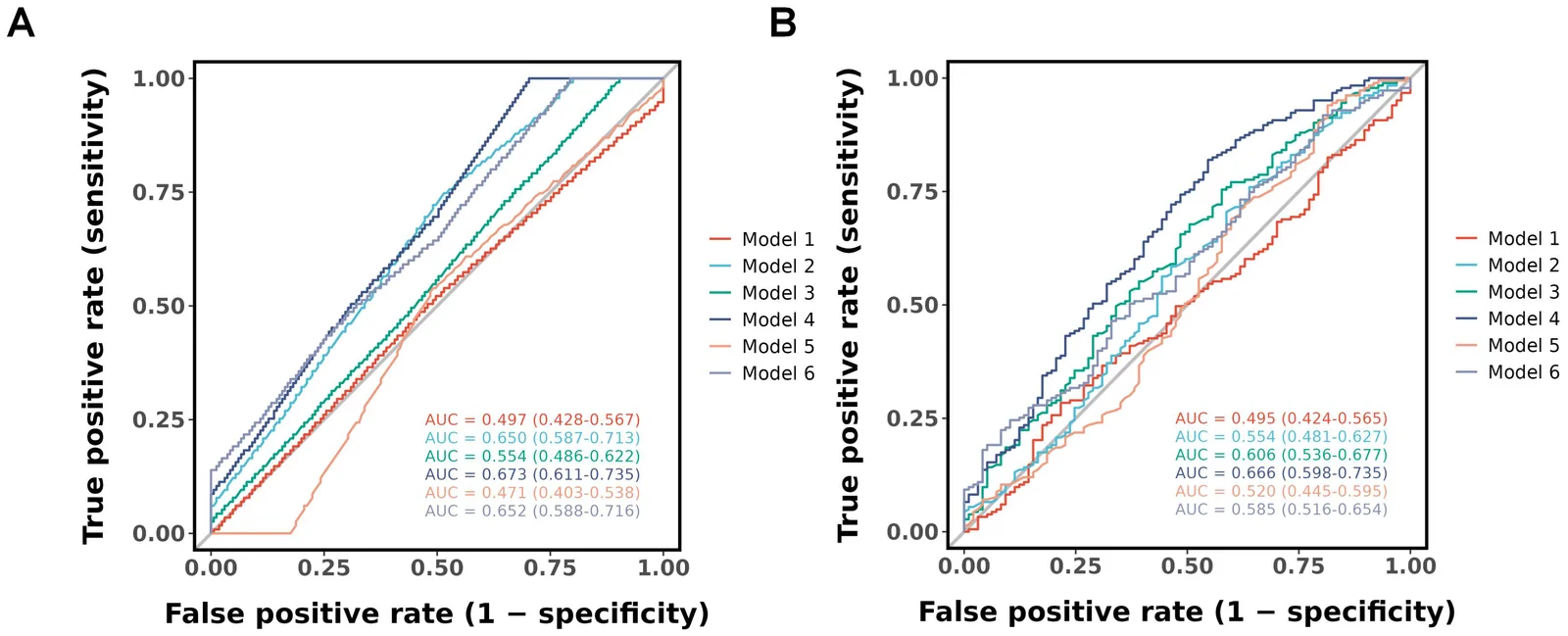
ROC curves of systemic inflammatory indices for pathological response. **(A)** ROC curves for pathological complete response. **(B)** ROC curves for favorable Miller–Payne pathological response. AUC values were used to compare the discriminatory ability of PLR, NLR, LMR, SII, SIRI, and PIV. ROC, receiver operating characteristic; AUC, area under the curve; pCR, pathological complete response.

The discriminatory ability of pretreatment inflammatory indices for pathological response was evaluated using receiver operating characteristic curve analysis, with detailed AUC values presented in **Table 6** and ROC curves shown in **Figure 3**. For pathological complete response (pCR), SII showed the highest AUC among the evaluated indices, with an AUC of 0.673 (95% CI: 0.611–0.735; cut-off 901.10). NLR and PIV demonstrated comparable but modest discriminatory ability, with AUCs of 0.650 (95% CI: 0.587–0.713; cut-off 2.39) and 0.652 (95% CI: 0.588–0.716; cut-off 475.90), respectively. LMR showed limited discriminatory performance, with an AUC of 0.554 (95% CI: 0.486–0.622; cut-off 2.50), whereas SIRI and PLR showed little discriminatory value, with AUCs of 0.471 (95% CI: 0.403–0.538; cut-off 0.87) and 0.497 (95% CI: 0.428–0.567; cut-off 160.39), respectively.

**Table 6:** ROC analysis of systemic inflammatory indices for pathological response endpoints.

Variable	AUC	95%CI	Cut-off value
Pathological complete response			
PLR	0.497	(0.428-0.567)	160.39
NLR	0.650	(0.587-0.713)	2.39
LMR	0.554	(0.486-0.622)	2.50
SII	0.673	(0.611-0.735)	901.10
SIRI	0.471	(0.403-0.538)	0.87
PIV	0.652	(0.588-0.716)	475.90
Favorable pathological response			
PLR	0.495	(0.424-0.565)	190.21
NLR	0.554	(0.481-0.627)	2.27
LMR	0.606	(0.536-0.677)	3.61
SII	0.666	(0.598-0.735)	802.81
SIRI	0.520	(0.445-0.595)	1.49
PIV	0.585	(0.516-0.654)	176.07

For favorable pathological response, SII again showed the highest AUC, with a value of 0.666 (95% CI: 0.598–0.735; cut-off 802.81). LMR and PIV demonstrated modest discriminatory ability, with AUCs of 0.606 (95% CI: 0.536–0.677; cut-off 3.61) and 0.585 (95% CI: 0.516–0.654; cut-off 176.07), respectively. NLR showed limited discriminatory performance, with an AUC of 0.554 (95% CI: 0.481–0.627; cut-off 2.27). SIRI and PLR showed little discriminatory value, with AUCs of 0.520 (95% CI: 0.445–0.595; cut-off 1.49) and 0.495 (95% CI: 0.424–0.565; cut-off 190.21), respectively. Overall, SII showed the highest, although modest, discriminatory ability for both pathological response endpoints among the evaluated inflammatory indices.

## Discussion

In this retrospective cohort, pretreatment inflammatory indices showed different associations with pathological response endpoints in patients with breast cancer receiving neoadjuvant therapy. Lower NLR, SII, and PIV were associated with a higher probability of pCR. Lower SII and PIV and higher LMR were associated with favorable Miller–Payne response. These findings suggest that different inflammatory indices may not behave identically across pathological endpoints.

Among the evaluated indices, SII showed the most consistent results. SII was associated with both pCR and favorable Miller–Payne response. However, its discriminatory ability was limited. The AUC values for SII were 0.673 for pCR and 0.666 for favorable Miller–Payne response. These values indicate modest discrimination rather than strong prediction. Therefore, SII should not be used alone to guide clinical decisions. It may be more appropriate as a supplementary marker when interpreted together with molecular subtype, HER2 status, Ki-67 index, tumor burden, and treatment regimen.

The difference between pCR and Miller–Payne response may explain part of the observed endpoint-specific findings. pCR reflects the complete disappearance of invasive disease, whereas Miller–Payne grading reflects the degree of reduction in tumor cellularity after treatment. These two endpoints may capture different aspects of treatment response. This may partly explain why NLR was associated with pCR but not with favorable Miller–Payne response, while LMR was associated with favorable Miller–Payne response but not with pCR.

The association between systemic inflammation and treatment response is biologically plausible. Chronic inflammation can promote tumor progression through tumor cell proliferation, angiogenesis, invasion, metastasis, and immune suppression (14, 15). Neutrophils and platelets are involved in these processes. Neutrophils may promote angiogenesis and suppress antitumor immunity, whereas platelets can protect circulating tumor cells and facilitate metastatic spread (19, 20). In contrast, lymphocytes are important for antitumor immune surveillance. A higher NLR, SII, or PIV may therefore reflect a systemic state with relatively stronger pro-tumor inflammation and weaker antitumor immunity.

LMR showed a different pattern. LMR was associated with favorable Miller–Payne response, but its association with pCR was not stable. This result may indicate that LMR reflects another dimension of host immune status. A higher LMR may represent relatively preserved lymphocyte-mediated antitumor immunity and lower monocyte-related inflammatory activity. Monocytes can contribute to tumor-associated macrophage polarization and the formation of an immunosuppressive microenvironment (21–23). This mechanism may explain why LMR was more closely related to substantial tumor regression than to complete pathological eradication.

Our findings are broadly consistent with previous studies on inflammatory markers in breast cancer. Several studies have reported associations between peripheral blood-derived inflammatory indices and response to neoadjuvant therapy or prognosis (16–18). However, previous findings have not been entirely consistent, and some studies did not demonstrate a significant or independent association between pretreatment inflammatory indices and pathological response (26). Differences in patient populations, molecular subtypes, treatment regimens, and marker definitions may partly explain these inconsistent results. However, many previous studies focused on one or a limited number of markers. In contrast, the present study compared several composite indices in the same cohort and assessed both pCR and Miller–Payne response. This design allowed us to observe that SII and PIV showed relatively stable associations, whereas other indices differed by endpoint.

Sensitivity analyses supported the main findings. After additional adjustment for relevant covariates, NLR, SII, and PIV remained associated with pCR. SII, PIV, and LMR remained associated with favorable Miller–Payne response. Similar patterns were observed when inflammatory indices were converted into categorical variables. These results suggest that the associations were not entirely dependent on one specific modeling strategy. However, these analyses should still be interpreted cautiously because multiple inflammatory indices and pathological endpoints were evaluated.

The age-stratified analyses yielded heterogeneous findings. Significant associations were observed in both age groups; however, formal interaction analyses identified evidence of effect modification only for PLR, with P values for interaction of 0.005 for pCR and 0.006 for favorable Miller–Payne response. The interaction terms for the other inflammatory indices were not statistically significant. Given the relatively small number of patients aged ≥60 years and the multiple subgroup comparisons, these findings should be considered exploratory.

From a clinical perspective, these indices are inexpensive and readily available from routine pretreatment blood counts. However, statistically significant associations do not necessarily indicate sufficient clinical usefulness. The absolute differences between response groups were relatively modest, and the distributions of these indices overlapped. For example, the median NLR values were 2.02 and 2.37 in the pCR and non-pCR groups, respectively, while the corresponding median SII values were 527 and 667 and the median PIV values were 248 and 311. Together with AUC values below 0.70, these findings indicate limited discriminatory ability at the individual-patient level. In addition, the ROC cut-off values were derived from the present cohort and varied according to the pathological endpoint. They should therefore be regarded as exploratory data-derived thresholds rather than clinically validated cut-off values. The current results do not support using these indices alone to predict pathological response, select patients for neoadjuvant therapy, or modify treatment regimens. They may instead be further evaluated as supplementary markers alongside established clinicopathological and molecular factors (24, 25).

This study has several limitations. First, its single-center retrospective design may have introduced selection bias and limits the generalizability of the findings. Second, patients received heterogeneous neoadjuvant treatment regimens, and differences in treatment intensity, platinum use, anti-HER2 therapy, and treatment completion may have influenced pathological response. Third, although relevant clinicopathological and treatment-related variables were considered, residual and unmeasured confounding cannot be excluded. Fourth, the inflammatory indices were derived from a single pretreatment blood measurement and are not tumor-specific; subclinical infection, metabolic status, medication use, and other systemic conditions may have affected peripheral blood cell counts. Fifth, multiple inflammatory indices, two pathological endpoints, and several sensitivity and subgroup analyses were evaluated, increasing the possibility of chance findings. The age- and subtype-specific analyses also had limited statistical power because of the relatively small sample sizes in some subgroups. Therefore, the age- and subtype-specific findings should be regarded as exploratory. Sixth, the ROC cut-off values were derived and evaluated in the same cohort and were not externally validated, which may have resulted in optimistic estimates of discriminatory performance. Finally, all individual AUC values were below 0.70, indicating limited discriminatory ability for standalone clinical prediction. Whether these inflammatory indices provide incremental predictive value beyond established clinicopathological and molecular factors remains uncertain and requires evaluation in larger independent prospective cohorts.

## Conclusion

In this retrospective study of 280 patients with breast cancer treated with neoadjuvant therapy, pretreatment inflammatory status showed a measurable association with pathological response. Lower NLR, SII, and PIV were related to a higher probability of pCR, while lower SII and PIV and higher LMR were associated with favorable Miller–Payne response. Among the evaluated indices, SII appeared to be the most stable marker across the two endpoints, but its AUC remained modest, indicating that it should not be used alone for clinical decision-making. These blood-based indices may be useful as simple supplementary markers, especially when interpreted together with molecular subtype, HER2 status, Ki-67, tumor burden, and treatment regimen. Because this was a single-center retrospective study without external validation, the findings should be regarded as exploratory. Larger prospective studies are needed to confirm their clinical value and to determine whether adding inflammatory indices can improve existing prediction models.

## Data Availability

The original contributions presented in the study are included in the article/**Supplementary Material**. Further inquiries can be directed to the corresponding author.
