# Supplementary material for: Associations between pretreatment composite inflammatory indices and pathological response to neoadjuvant therapy in breast cancer: a retrospective cohort study
Source: Front Oncol. 2026 Aug 17;16:1913071. doi: 10.3389/fonc.2026.1913071 (PMC13527441; doi:10.3389/fonc.2026.1913071)
Supplement: Supplementary file 1 [file Table1.docx]

**Supplementary Table1. Age-related interaction analysis between inflammatory indices and pathological response endpoints**

| **Inflammatory index** | **P for interaction** | |
| --- | --- | --- |
|  | **Pathological complete response** | **Favorable Miller–Payne response** |
| PLR | 0.005 | 0.006 |
| NLR | 0.490 | 0.178 |
| LMR | 0.252 | 0.713 |
| SII | 0.443 | 0.674 |
| SIRI | 0.297 | 0.939 |
| PIV | 0.618 | 0.269 |

Abbreviations: pCR, pathological complete response; PLR, platelet-to-lymphocyte ratio; NLR, neutrophil-to-lymphocyte ratio; LMR, lymphocyte-to-monocyte ratio; SII, systemic immune-inflammation index; SIRI, systemic inflammation response index; PIV, pan-immune-inflammation value.
